# Integrated metagenomic and metabolomic insights into microbial metabolic reprogramming in the rhizosphere of the invasive plant *Praxelis clematidea* under low-temperature stress

**DOI:** 10.3389/fmicb.2026.1852122

**Published:** 2026-07-15

**Authors:** Xiaowen Liu, Wenzhi Cheng, Chunhong Li, Wubliker Dessie, Chengmei Qi, Muhammad Ayaz, Xiangqin Xu

**Affiliations:** 1 College of Chemistry and Bioengineering, Hunan University of Science and Engineering, Yongzhou, China; 2 Hunan Provincial Engineering Research Center for Green Control of Soil-borne Plant Diseases, Yongzhou, China; 3 Yongzhou City Radiation Environmental Supervision Station, Yongzhou, China; 4 Key Laboratory of Biopesticide Creation and Resource Utilization in Inner Mongolia Autonomous Region, College of Horticulture and Plant Protection, Inner Mongolia Agricultural University, Hohhot, China

**Keywords:** low-temperature stress, metabolomics, metagenomics, microbial community, *Praxelis clematidea*, rhizosphere microbial ecology

## Abstract

A primary factor preventing the spread of the invasive plant *Praxelis clematidea* to higher latitudes and altitudes is the low-temperature stress induced by global climate change. The present study investigated the impact of low-temperature stress on the rhizosphere soil micro-ecosystem of *P. clematidea*, with the aim of examining its adaptive micro-ecological mechanisms via a comprehensive multi-omics approach. The rhizosphere soils of plants were compared under low-temperature (LT, 5 °C) or normal-temperature (HT, 25 °C) treatments. Using soil physicochemical analysis, enzyme activity assay, metagenomics, and non-targeted metabolomics, we observed that LT stress did not significantly alter microbial alpha diversity but strongly shifted the community structure. This change enriched cold-tolerant bacterial taxa, including *Nocardiopsis, Sphingobium* and *Azoarcus.* The LT stress was associated with altered carbon and nitrogen cycling, as indicated by increased soil urease activity but decreased alkaline phosphatase and catalase activities. The nitrate-N and ammonium-N levels increased, but total nitrogen, total organic carbon, and organic matter were reduced. Additionally, metagenomic study revealed overexpression of major microbial carbon metabolism genes (e.g., TCA cycle and glycolysis) and downregulation of nitrogen assimilation genes (e.g., *glnA* and *NasA*). Furthermore, metabolomics indicated a rise in carbohydrates and vitamins, along with a notable accumulation of stress-resistant secondary metabolites such as phenolic acids, flavonoids, and terpenes in the rhizosphere soils under LT stress. Correlation analysis indicated strong positive associations between the enriched cold-tolerant genera and these stress-resistant metabolites (e.g., costunolide and choline sulfate). Functional enrichment analysis suggested a metabolic reprogramming signature coupled with low-temperature treatment. Finally, this integrated multi-omics study reveals that *P. clematidea* is associated with an altered rhizosphere microbiome, differential functional gene abundance, and reorganized metabolic networks under low-temperature conditions. These findings offer a vital micro-ecological elucidation for *P. clematidea* effective colonization and propagation in novel, colder habitats.

## Graphical abstract

**Figure:**
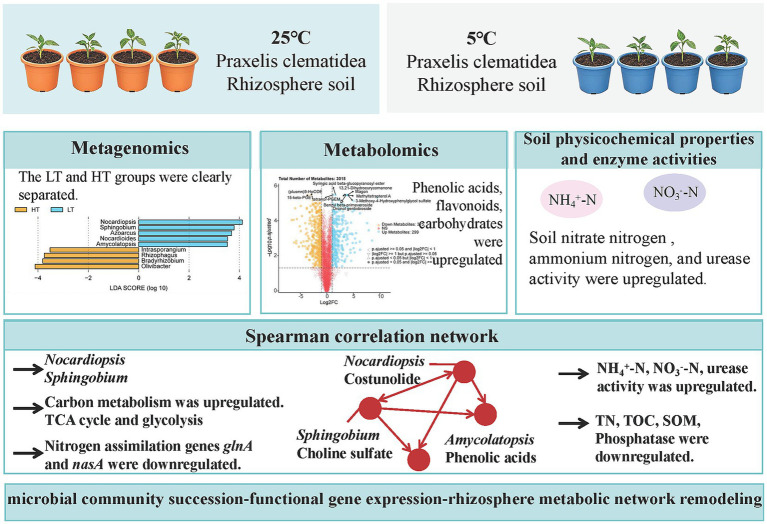
The graphical abstract provides an integrated visual summary of the experimental design, core analytical approaches, and key mechanistic findings of this study.

## 1 Introduction

The invasive species *P. clematidea*, originally from South America, has proliferated in southern China due to its robust adaptability and competitive ability, thereby threatening local biodiversity and agricultural production (Liang et al., 2023). The previous study has concentrated on the invader’s life-history such as rapid development and extensive reproductive output and its above-ground interactions, including allelopathy (Vercaemer et al., 2021; Wu et al., 2021; Yuan et al., 2020). However, with ongoing global climate change and the potential of *P. clematidea* to expand into higher latitudes and altitudes, low-temperature stress has emerged as a critical environmental factor limiting its further poleward expansion (Wang J. G. et al., 2025). Its remarkable adaptability and rapid dispersal enable successful colonization across diverse environments, and understanding its response to low-temperature stress in non-native ranges is key to explaining its invasion success (Fu et al., 2017; Zhou et al., 2017). The rhizosphere, the interface of plant–soil-microbe interactions, represents the most active zone for biogeochemical cycling in soil (He et al., 2024; Wang et al., 2023). This micro-ecosystem plays a vital role in plant adaptation to environmental stress by modulating soil nutrient availability, enhancing plant stress resistance, and influencing community structure (Kong and Liu, 2022; Neemisha et al., 2022). Recent advances in holobiont theory conceptualize the plant and its associated microbiome as a single ecological and evolutionary unit, wherein the rhizosphere microbiota functions as an extended phenotype that contributes to host fitness under environmental stress (Manzanera, 2025; Vandenkoornhuyse et al., 2015). Understanding how low-temperature stress reshapes this plant–microbe meta-organism is therefore crucial for elucidating the invasion success of P. clematidea in colder habitats.

Low-temperature stress alters the rhizosphere microenvironment by influencing plant physiological and biochemical processes, as well as modifying soil physicochemical properties and microbial activity (Parasar et al., 2024). Under low-temperature conditions, key nutrient cycles, such as carbon, nitrogen, and phosphorus, undergo significant changes (Khan et al., 2021). For instance, low temperatures can inhibit microbial enzyme activities, thereby affecting the decomposition of organic matter and nutrient mineralization processes (Chen and Shiau, 2025). To cope with cold stress, however, plants may adjust the composition of their root exudates, which in turn can recruit or select for specific cold-tolerant microbial communities to help maintain nutrient uptake and enhance stress resistance (Kazarina et al., 2024; Zeng et al., 2024). The rhizosphere microbial community plays an indispensable role in plant nutrient cycling. These microorganisms directly or indirectly regulate plant acquisition of nutrients like nitrogen and phosphorus by secreting extracellular enzymes and participating in processes such as nitrogen fixation, nitrification, denitrification, and phosphate solubilization (Revillini et al., 2024). For example, soil urease (S-UE) activity is closely associated with nitrogen mineralization (Zhang et al., 2023), while soil alkaline phosphatase (S-ALPT) is crucial for the mobilization of organic phosphorus (Li et al., 2021). The changes in the activities of these enzymes under low temperatures directly impact soil nutrient availability. Concurrently, shifts in microbial community structure, particularly the enrichment of cold-adapted taxa, are considered a key strategy for plant adaptation to low temperatures. The specific bacteria including *Enterobacter* sp., and arbuscular mycorrhizal fungi (AMF), like those from the genus *Rhizophagus*, can form symbiotic relationships with plants, enhancing their nutrient acquisition and stress resilience (Sujkowska-Rybkowska et al., 2023; Wahab et al., 2023). Despite extensive documentation of microbial community shifts under abiotic stress, few studies have simultaneously integrated metagenomic and metabolomic approaches to mechanistically link specific cold-enriched taxa to functional gene expression patterns and the accumulation of stress-resistant metabolites in the rhizosphere of invasive plants. This knowledge gap limits our ability to understand how the plant holobiont coordinates below-ground responses to cold stress.

The current work aims to explore the processes by which low-temperature induction affects *P. clematidea* rhizosphere soil micro-ecosystem with the goal to better understand how the plant sustains its ecological niche under low-temperature stress. Using a controlled environment with low-temperature (LT, 5 °C) and normal-temperature (HT, 25 °C) treatments, we integrated soil physicochemical analysis, enzyme activity assays, metagenomics, metabolomics, and multi-omics correlation analysis. We hypothesized that low-temperature stress acts as a strong environmental filter that selectively enriches cold-adapted microbial taxa, which in turn drive coordinated shifts in carbon and nitrogen metabolism gene abundance and metabolite accumulation, collectively contributing to enhanced rhizosphere stress tolerance.

## 2 Materials and methods

### 2.1 Sample collection and experimental design

The soil samples utilized in this study were collected in October 2024 from farmland near Xiangling Mountain (E 111°37′, N 26°14′), Lingling District, Yongzhou City, Hunan Province, China. This region experiences a subtropical monsoon climate, and the sampling site was a typical area invaded by *P. clematidea*. Healthy *P. clematidea* plants with uniform growth were selected. Rhizosphere soil, defined as soil tightly adhering to the root surface and strongly influenced by root activities, was collected using the “shaking-root method.” The rhizosphere soil samples from multiple points were thoroughly mixed to form a homogenized initial soil sample, which was then passed through a 2-mm sieve to remove plant debris and stones. Three independent biological replicates were established for each treatment (*n* = 3), with each replicate representing the rhizosphere soil of a single *P. clematidea* plant grown in a separate pot. No sample pooling was performed across replicates. All downstream analyses—soil physicochemical measurements, enzyme activity assays, metagenomic sequencing, and metabolomic profiling—were conducted independently on each of the three replicates per treatment group.

A pot-based controlled experiment was conducted. The homogenized soil was placed into uniform plastic pots (upper diameter × height × bottom diameter = 15 cm × 14 cm × 11 cm), with each pot containing 1.5 kg of soil. One pre-grown *P. clematidea* seedling (approximately 10 cm in height) with consistent growth, cultivated in the same soil, was transplanted into each pot. All potted plants were pre-cultured in a growth chamber at 25 °C, 70% relative humidity, and a 12 h/12 h (light/dark) photoperiod for two weeks to acclimate and resume growth. After pre-cultivation, the pots were randomly divided into two groups: a low-temperature treatment group (LT group) and a normal-temperature control group (HT group). The LT group was transferred to an artificial climate chamber set at 5 °C for low-temperature induction, while the HT group remained at 25 °C. Other environmental conditions (light and humidity) for both groups were maintained consistent with the pre-cultivation phase. The treatment period lasted for 8 weeks. During this time, pots were regularly weighed and replenished with deionized water to maintain stable soil moisture content. At the end of the treatment, rhizosphere soil was collected from each pot using the same method as the initial sampling.

The rhizosphere soil samples within the same treatment group were pooled to obtain representative composite samples. The samples were quickly passed through a 2-mm sieve and divided into three parts: one part of fresh soil was stored at 4 °C for determining soil water content, microbial biomass carbon, and soil enzyme activities (all analyses were completed within one week); another part of fresh soil was stored at −80 °C for subsequent soil metagenomic and metabolomic analyses; the remaining soil was air-dried, ground, and passed through sieves of different mesh sizes for the determination of basic soil physicochemical properties.

### 2.2 Soil physicochemical properties and enzyme activities analysis

Soil physicochemical properties and enzyme activities were determined following established methods (Rubaiyat et al., 2023; Wang et al., 2022). The soil pH was measured potentiometrically using a composite electrode with a soil-to-water ratio of 1:2.5. The soil organic matter (SOM) content was determined by the potassium dichromate volumetric method. The total nitrogen (TN) was measured using the semi-micro Kjeldahl method. Total phosphorus (TP) was determined by colorimetry after digestion with HClO₄-H₂SO₄. Available phosphorus (AP) was extracted with NaHCO₃ and measured by the molybdenum-antimony anti-colorimetric method. Nitrate-nitrogen (NO₃^−^-N) and ammonium-nitrogen (NH₄^+^-N) were extracted with 0.01 mol L^−1^ CaCl₂ and analyzed using a continuous flow analyzer (AA3, SEAL Analytical, Germany). The total organic carbon (TOC) was quantified by the potassium dichromate oxidation-external heating method. The microbial biomass carbon (MBC) was determined by the chloroform fumigation-K₂SO₄ extraction method. S-UE activity was assayed using the sodium phenolate-sodium hypochlorite colorimetric method. The S-ALPT activity was measured by the disodium phenyl phosphate colorimetric method while soil catalase (S-CAT) activity was determined by potassium permanganate titration.

### 2.3 Metagenomic sequencing and data analysis

The total genomic DNA was extracted from approximately 0.5 g of fresh rhizosphere soil (stored at −80 °C) using the Cetyltrimethylammonium bromide (CTAB) method. The purity and concentration of the extracted DNA were assessed using a NanoDrop™ One spectrophotometer (Thermo Fisher Scientific, United States) and a Qubit® 4.0 fluorometer (Invitrogen, United States), respectively, while DNA integrity was evaluated via 1% agarose gel electrophoresis. Qualified DNA samples were sent to Shenzhen Microeco Bio Ltd. for metagenomic sequencing on the Illumina NovaSeq 6,000 platform (Illumina, United States). Sequencing libraries with an insert size of approximately 350 bp were constructed and subjected to paired-end 150 bp (PE150) sequencing. The raw sequencing data were first processed using Fastp (v0.23.1) for quality control, which involved removing adapter sequences, low-quality bases (Q < 20), and reads with an excessive proportion of N bases, resulting in high-quality clean data. Subsequent bioinformatic analyses were performed on the Shengkeyun Bioincloud platform.^1^ High-quality clean data from each sample were assembled *de novo* using MEGAHIT (v1.2.9) to generate scaffolds. Open Reading Frames (ORFs) were predicted from the assembly results using MetaGeneMark (v3.38). The predicted amino acid sequences (length ≥ 100 bp) were compared against the NCBI NR database using Diamond (v2.0.9) for taxonomic annotation. Based on the assembly, clean reads from each sample were mapped to the non-redundant gene catalog using Bowtie2 (v2.4.1), and gene abundance was estimated using Salmon (v1.5.2) to generate taxonomic and functional abundance profiles. Gene abundance was quantified as transcripts per million (TPM) and normalized by total mapped reads per sample.

Alpha diversity indices (including ACE and Shannon) were calculated, and beta diversity was analyzed using Non-metric Multidimensional Scaling (NMDS) based on Bray-Curtis distances, implemented in MOTHUR (v1.43.0) and the R vegan package (v4.1.3). The linear Discriminant Analysis Effect Size (LEfSe) analysis, with an LDA score threshold set to 3.5—a stringent cutoff commonly used to identify highly discriminative features with biological significance—was used to identify differentially abundant biomarkers at the genus level. FDR-adjusted *p*-values were also reported for all LEfSe results. Functional annotation was performed using the KEGG Orthology (KO) database. Differential abundance analysis of key functional genes involved in carbon and nitrogen metabolism was conducted and visualized using heatmaps in STAMP software (v2.1.3). The spearman’s rank correlation coefficient was used to analyze correlations between taxa/functional genes and soil physicochemical properties, and the results were visualized using the corrplot package in R language.

### 2.4 Untargeted metabolomics analysis by LC-MS/MS

Briefly, 0.1 g of rhizosphere soil sample was resuspended in pre-chilled 80% methanol by vigorous vortexing. The soil extract was then incubated on ice for 5 min, followed by centrifugation at 15,000 × g for 20 min at 4 °C. A portion of the supernatant was diluted with LC–MS grade water to a final methanol concentration of 53%. The diluted sample was transferred to a new tube and centrifuged again at 15,000 × g for 20 min at 4 °C. The resulting supernatant was injected into the LC–MS/MS system for analysis.

Metabolomic profiling was conducted through an ultra-high-performance liquid chromatography (UHPLC) system coupled with a triple quadrupole time-of-flight (TOF) mass spectrometer (Waters, Milford, MA, United States). Metabolite separation was achieved on a HILIC TSK gel Amide-80 column (2.0 mm × 250 mm, 5 μm; Tosoh Bioscience, Tokyo, Japan) equipped with a guard column of the same material (2.0 mm × 1 cm). The flow rate was set at 0.15 mL/min using a gradient elution with solvent A (acetonitrile) and solvent B (3 mM ammonium acetate in water, pH adjusted to 5.5 with acetic acid) as follows: 0–3 min, 5% B isocratic; 3–7 min, 5% B to 70% B linear gradient; 27–30 min, 70% B isocratic; 30–32 min, 70% B to 5% B back to initial conditions; 32–40 min, 5% B isocratic for column re-equilibration. The mass spectrometer was operated in positive electrospray ionization (ESI) full-scan mode. Spectral peaks were automatically calibrated for locked mass fluctuation. Raw data files were converted to mzML format using MSConvert in ProteoWizard. The processed LC–MS raw data were used for metabolite identification against the Metlin database.

The scaled and normalized data were imported into R software (using the Ropls package) for multivariate analysis, including Principal Component Analysis (PCA) and Orthogonal Projections to Latent Structures-Discriminant Analysis (OPLS-DA). A Support Vector Machine (SVM) model was applied to identify features contributing most significantly to inter-group differences. Differential metabolites were identified by applying the following criteria: fold change (FC > 1.5), Variable Importance in Projection (VIP > 1) from the OPLS-DA model, and a Benjamini-Hochberg false discovery rate (FDR)-adjusted *p*-value < 0.05. A specific set of metabolites selected from the OPLS-DA were subjected to Over-Representation Analysis (ORA) to identify key KEGG pathways significantly enriched with these metabolites, and topological influence was also calculated. All OPLS-DA models were validated using permutation tests (*n* = 200), and the corresponding R^2^Y and Q^2^ values are reported in Supplementary information.

### 2.5 Dominant microbial genera and differential metabolites integrated analysis

To obtain a greater knowledge of the interactions between the microbial community and metabolic functions in the rhizosphere micro-ecosystem of *P. clematidea* under low-temperature stress, we conducted a comprehensive correlation analysis of metagenomic data (relative abundance of microbial genera) and untargeted metabolomics data (Differentially Expressed Metabolites, DEMs). All analyses were based on three independent biological replicates (*n* = 3).

The Orthogonal Projections to Latent Structures-Discriminant Analysis (OPLS-DA) was applied separately to the microbial genera, functional genes (KEGG Orthology, KO), and metabolite datasets for pattern recognition, aiming to identify variables with the greatest contribution to distinguishing between the LT and HT treatment groups (VIP > 1.0, *p* < 0.05). Based on these criteria, the top ten differential microbial genera, key functional genes, and differential metabolites (DMs) were extracted from each omics dataset for subsequent correlation analysis. Subsequently, three separate correlation heatmaps were constructed based on Spearman’s rank correlation coefficients. The significance threshold for correlations was set at |r| > 0.6 with *p* < 0.05. All heatmaps were visualized using the pheatmap package in R (v4.2.0), with the color gradient representing the magnitude of the correlation coefficient (red representing positive correlation, blue indicating negative correlation).

## 3 Results

### 3.1 Low-temperature effect soil physicochemical properties and enzyme activities

As shown in Table 1, compared to the HT, the LT significantly altered the micro-ecological environment of the *P. clematidea* rhizosphere soil, inducing substantial changes in both its physicochemical properties and key enzyme activities. Significant effects of low-temperature treatment were observed on soil enzyme activities. The S-UE activity in the LT group was significantly higher than that in the HT group (*p* < 0.05). In contrast, the activities of both S-ALPT and catalase (S-CAT) in the LT group were significantly lower than those in the HT group (*p* < 0.05).

**Table 1:** Comparison of rhizosphere soil physicochemical properties and enzyme activities of *Praxelis clematidea* under different temperature treatments.

Group	S-UE (μg/d/g)	S-ALPT (mg/d/g)	S-AKP (μmol/d/g)	Nitrate_N (mg/kg)	Ammonium_N (mg/kg)	S-CAT (mmol/d/g)	TOC (g/kg)	MBC (g/kg)	OM (g/kg)	TN (g/kg)	TP (g/kg)	AP (mg/kg)
HT (25 °C)	208.44 ± 1.76 b	0.42 ± 0.02 a	5.91 ± 0.25 a	0.19 ± 0.06 b	0.03 ± 0.02 b	27.21 ± 0.79 a	42.67 ± 3.04 a	174.34 ± 66.32 a	70.58 ± 3.61 a	0.94 ± 0.05 a	0.28 ± 0.03 a	35.59 ± 4.08 a
LT (5 °C)	275.47 ± 6.21 a	0.26 ± 0.01 b	5.02 ± 0.03 b	0.70 ± 0.05 a	1.99 ± 0.06 a	24.22 ± 0.31 b	29.23 ± 1.82 b	160.45 ± 52.77 a	49.31 ± 2.66 b	0.46 ± 0.06 b	0.44 ± 0.09 a	25.58 ± 2.11 b

Different letters within the same column indicate significant differences between treatments at the *p* < 0.05 level.

The LT distinctly altered the forms and distribution of soil nitrogen. The contents of NO₃^−^-N and NH₄^+^-N in the LT group rhizosphere soil were significantly higher than those in the HT group (*p* < 0.05), with a particularly pronounced increase in NH₄^+^-N content. Likewise, in comparison with the trend noted for inorganic nitrogen, the total nitrogen (TN) content in the low-temperature (LT) group was considerably lower than that in the high-temperature (HT) group (*p* < 0.05). Regarding the soil carbon pool and related indicators, the contents of TOC and SOM in the LT group were significantly lower than those in the HT group (*p* < 0.05). In contrast, MBC showed no significant difference between the two treatment groups (*p* > 0.05). For phosphorus, although the TP content did not show a significant difference between treatments (*p* > 0.05), the available phosphorus (AP) content in the LT group was significantly lower than that in the HT group (*p* < 0.05).

### 3.2 Low-temperature impact on rhizosphere soil microbial community

**Figure 1:**
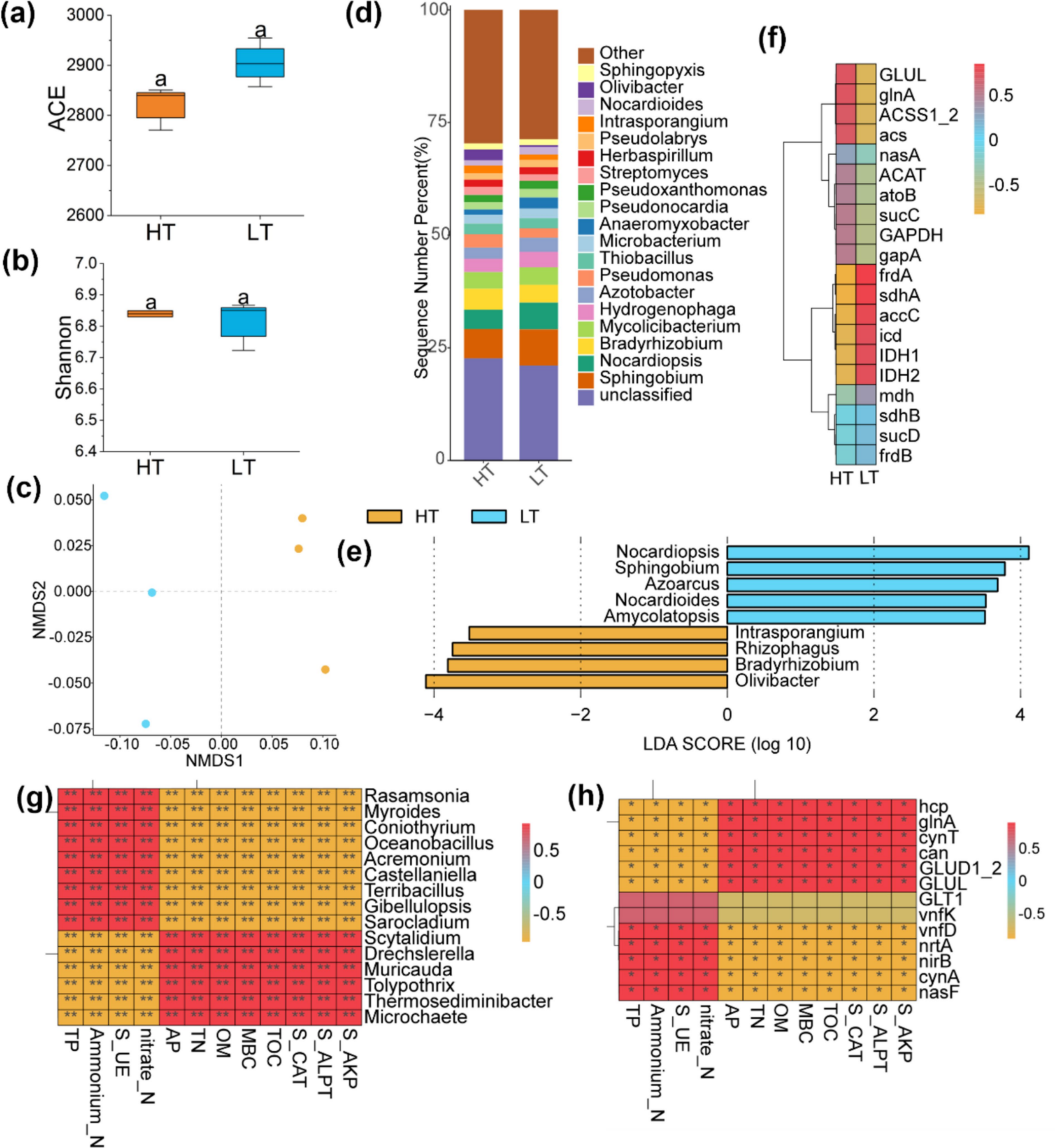
Effects of low temperature treatment on the rhizosphere soil microbial community and function of the invasive plant *Praxelis clematidea*. **(a,b)** ACE index **(a)** and Shannon index **(b)** of the rhizosphere soil microbial community of *Praxelis clematidea* under different temperature treatments. HT: 25 °C normal temperature treatment group; LT: 5 °C low temperature treatment group. Different lowercase letters indicate significant differences between treatments at *p* < 0.05 level (*n* = 3). Error bars represent standard deviation. **(c)** Non-metric multidimensional scaling (NMDS) analysis based on Bray-Curtis distances showing differences in microbial community structure between normal temperature (HT) and low temperature (LT) treatments. **(d)** Key taxonomic composition of rhizosphere microorganisms at the genus level under different treatments. **(e)** LEfSe analysis identifying significantly differentiated biomarkers at the genus level. The figure shows the significantly differential biomarkers (at genus level) with an LDA score greater than 3.5. **(f)** Heatmap displaying differential abundance of key functional genes involved in carbon and nitrogen metabolism between the two treatment groups. **(g)** Heatmap of correlation analysis between microbial species and soil physicochemical properties. The color scale represents Z-score normalized values of gene abundance, with red indicating up-regulation and blue indicating down-regulation. **(h)** Heatmap of correlation analysis between functional genes and soil physicochemical properties.

A detailed analysis of metagenomic data from the rhizosphere soil of P. clematidea under low-temperature (LT) and high-temperature (HT) treatments indicated significant effects of low-temperature stress on the rhizosphere micro-ecology across various dimensions. The main results are summarized in Figure 1. The results indicated no significant differences in either the ACE index (Figure 1a) or the Shannon index (Figure 1b) between the LT and HT treatment groups (*p* > 0.05). Non-metric multidimensional scaling (NMDS) analysis showed clear spatial separation between the sample points of the HT and LT groups (Figure 1c), indicating that temperature treatment was a decisive factor driving the variation in rhizosphere microbial community structure (Bray-Curtis distance, Stress < 0.05). This demonstrates that while low-temperature induction altered the community structure of rhizosphere microorganisms, it did not significantly affect the overall alpha diversity. Further analysis of the composition structure of the rhizosphere soil microbial community at the genus level (Figure 1d) showed that it was primarily composed of genera such as *Sphingobium*, *Nocardiopsis*, *Bradyrhizobium*, and *Mycolicibacterium*, which collectively form the core microbiome of the *P. clematidea* rhizosphere. After low-temperature induction, the microbial community underwent a drastic and directional shift. The most notable change was the extreme enrichment of the relative abundances *of Nocardiopsis, Sphingobium, Azoarcus, Nocardioides*, and *Amycolatopsis* in the LT group. In contrast, the relative abundances of numerous species, including *Intrasporangium*, *Rhizophagus*, *Bradyrhizobium,* and *Olivibacter,* which were more prevalent in the HT group, were substantially decreased in the LT group. This suggests that low-temperature stress acted as a strong environmental filter on the rhizosphere microbial community of *P. clematidea*, selecting for cold-tolerant or psychrophilic microbial taxa with specific functional traits.

Analysis of the relative abundance of key functional genes involved in carbon and nitrogen metabolism revealed that low-temperature treatment was associated with distinct differences in the metabolic genetic potential of the rhizosphere microbial community of *P. clematidea*. Heatmap analysis (Figure 1f) displayed differential abundance of multiple key functional genes between the HT and LT treatment groups.

Regarding nitrogen metabolism, the *glnA* gene, encoding glutamine synthetase, showed a downregulation trend in the LT group, consistent with previous findings. Similarly, the *nasA* gene, associated with nitrate assimilation, exhibited a comparable pattern of change. For carbon metabolism, the low-temperature treatment significantly impacted several key pathways. Genes related to the tricarboxylic acid (TCA) cycle (*sdhA*, *sdhB*, *sucC*, *sucD*, *frdA*, *frdB*, and *mdh*) were generally upregulated in the LT group. The expression of key glycolytic pathway genes (*GAPDH*, *gapA*) was enhanced under LT conditions. Genes involved in acetate metabolism (*ACSS1_2*, *acs*) also showed an upregulation trend, while changes were observed in the expression of genes related to isocitrate metabolism (*icd*, *IDH1*, and *IDH2*). The correlation heatmap between microbial taxa and soil physicochemical properties (Figure 1g) revealed that genera significantly enriched in the LT group, such as *Nocardiopsis*, *Azoarcus*, and *Amycolatopsis*, showed significant positive correlations with soil NH₄^+^-N and NO₃^−^-N contents, but significant negative correlations with TN content. Furthermore, the relative abundance of *Sphingobium* was negatively correlated with TOC and SOM contents. The genera more strongly associated with the HT group, such as *Bradyrhizobium*, exhibited positive correlations with TN, TOC, and SOM contents. Regarding the phosphorus cycle, the abundance of genera like *Oceanobacillus* showed positive correlations with AP content and S-ALPT activity (*p < 0.05).

Further correlation analysis was performed between key functional genes for carbon/nitrogen cycling and soil physicochemical properties/enzyme activities (Figure 1h). The results indicated that nitrogenase genes (*vnfD* and *vnfK*) and the nitrite reductase gene (*nirB*) were significantly positively correlated with soil NH₄^+^-N and NO₃^−^-N contents. Conversely, the *glnA* gene (glutamine synthetase), the nitrate/nitrite transporter gene (*nrtA*), and the nitrate assimilation gene (*nasF*) showed significant positive correlations with soil TN content but significant negative correlations with NH₄^+^-N content. Cyanase genes (*cynA*, *cynT*) were positively correlated with S-UE activity. Regarding carbon cycle-related genes, the *hcp* gene, encoding a high-affinity urea active transporter, was positively correlated with TOC and SOM contents. Inversely, the carbonic anhydrase gene (*can*) exhibited a significant negative correlation with S-CAT activity. Additionally, the glutamate synthase gene (*GLT1*) was positively correlated with AP content.

### 3.3 Response of rhizospheric metabolites and metabolic pathways

**Figure 2:**
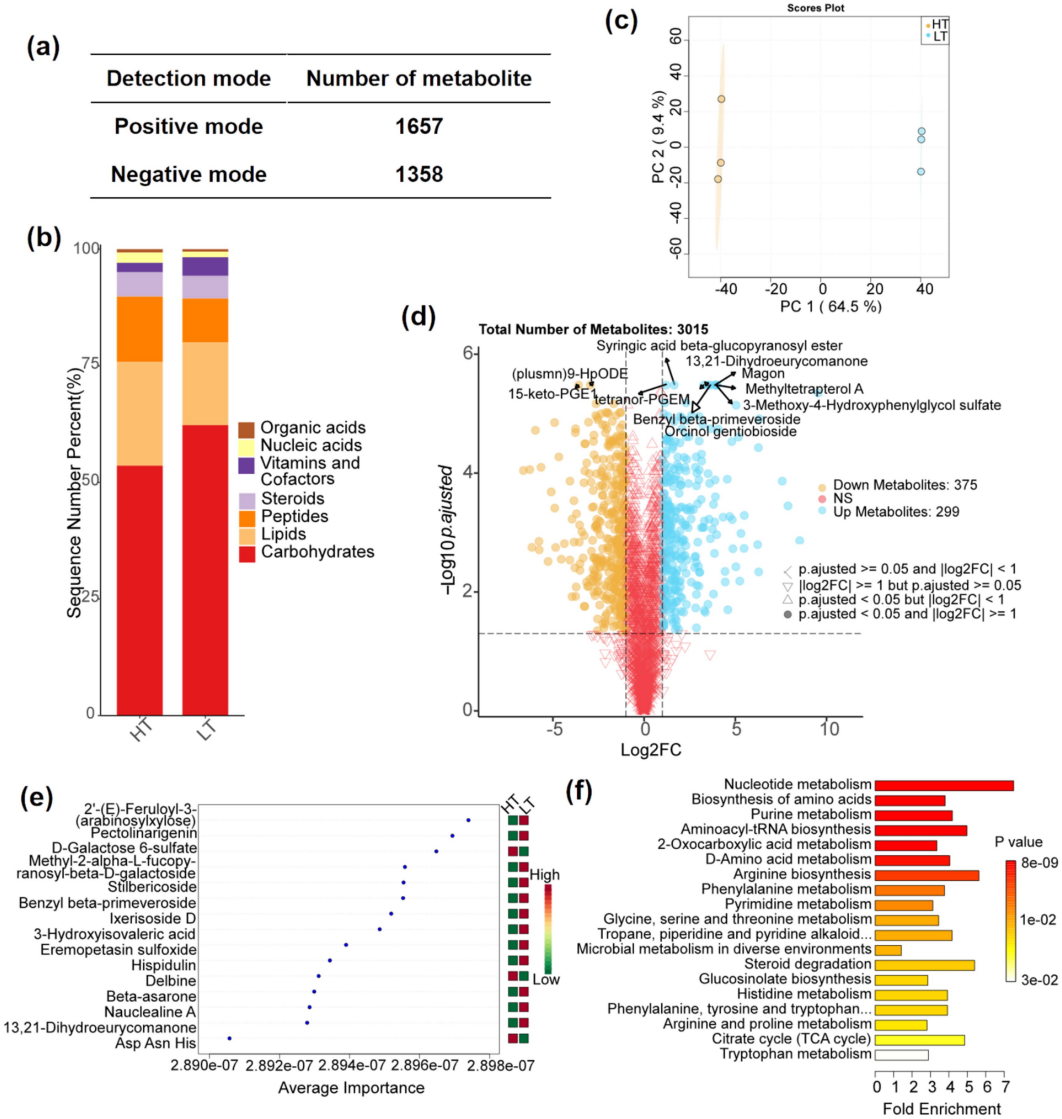
Non-targeted metabolomics analysis of the rhizosphere soil of *Praxelis clematidea* under low-temperature stress. **(a)** Statistics of the number of metabolites detected based on positive and negative ion modes. **(b)** Examples of the names of some representative metabolites identified in this study. **(c)** The number of differentially expressed metabolites (DEMs), both up-regulated and down-regulated, in the low-temperature stress group compared to the control group. NS indicates Not Significant. **(d)** Volcano plot of differentially expressed rhizosphere soil metabolites between treatments. **(e)** Volcano plot of differentially expressed metabolites in the rhizosphere soil of *P. clematidea* between low temperature and normal temperature treatments. Each point represents a metabolite. Red dots represent significantly up-regulated DEMs, blue dots represent significantly down-regulated DEMs and gray dots represent metabolites with no significant difference. (p.adj < 0.05 and Log2FC ≥ 1). **(f)** Pathway enrichment analysis of differentially expressed metabolites in the rhizosphere of *P. clematidea*. The size of the dots represents the fold enrichment, and the color depth represents the significance level of enrichment [−log10 (*p* value)].

An extensive examination of the rhizosphere soil metabolites of *P. clematidea* was carried out by untargeted metabolomics, resulting in the identification of 3,015 differential expression metabolites (DEMs) (Figure 2a). The functional annotation and classification of these DEMs yielded their relative percentage distribution across major metabolite classes (Figure 2b). The results indicated that low-temperature induction significantly altered the compositional profile of rhizosphere soil metabolites. The relative contents of carbohydrates, vitamins, and cofactors were significantly increased in LT. Conversely, the relative contents of lipids and peptides were significantly decreased. The changes in the relative contents of nucleic acids, organic acids, and steroids were relatively minor between the two groups. PCA was employed to assess the overall effect of low-temperature treatment on the metabolite composition of *P. clematidea* rhizosphere soil. The PCA results (Figure 2c) showed that PC1 and PC2 effectively distinguished the HT and LT treatment groups.

To properly screen and identify significant changes in important metabolites under low-temperature stress, we conducted a statistical analysis (t-test) on the untargeted metabolomics data and produced a volcano plot (Figure 2d). Among all 3,015 detected metabolites, 674 significant DEMs were identified, of which 299 metabolites were significantly upregulated and 375 were significantly downregulated in the LT group. Representative significantly upregulated metabolites are annotated in the figure, including Syringic acid beta-glucopyranosyl ester, 13,21-Dihydroeurycomanone, and Benzyl beta-primeveroside.

The Support Vector Machine-Recursive Feature Elimination (SVM-RFE) algorithm was employed for screening, effectively identifying critical biomarkers that optimally differentiated the two groups, as illustrated in (Figure 2e). This model successfully filtered a series of important metabolites with high discriminatory power. Compared to the HT group, phenolic acids (e.g., 2′-(E)-Feruloyl-3-(arabinosylxylose), Stilbericoside, Benzyl beta-primeveroside), flavonoids (e.g., Pectolinarigenin, Hispidulin), and terpenoids (e.g., 13,21-Dihydroeurycomanone, Eremopetasin sulfoxide, Beta-asarone) were significantly upregulated in the LT group, while glycoside derivatives (e.g., D-Galactose 6-sulfate) and amino acids (e.g., Asp., Asn, and His) were significantly downregulated, potentially serving as potential biomarkers (Figure 2e).

The ORA pathway enrichment analysis of the DEMs under low-temperature stress revealed their significant enrichment in several important metabolic pathways (Figure 2f). The enrichment results showed that Nucleotide metabolism, Biosynthesis of amino acids, and Purine metabolism were among the most significantly enriched pathways (*p* < 0.01). Moreover, pathways such as aminoacyl-tRNA biosynthesis, 2-Oxocarboxylic acid metabolism, and D-Amino acid metabolism also showed highly significant enrichment. Notably, pathways related to the synthesis of specific compounds, such as Tropane, piperidine and pyridine alkaloid biosynthesis and Glucosinolate biosynthesis, were also significantly enriched.

### 3.4 Linkages between rhizosphere microbiota and metabolites under LT stress

**Figure 3:**
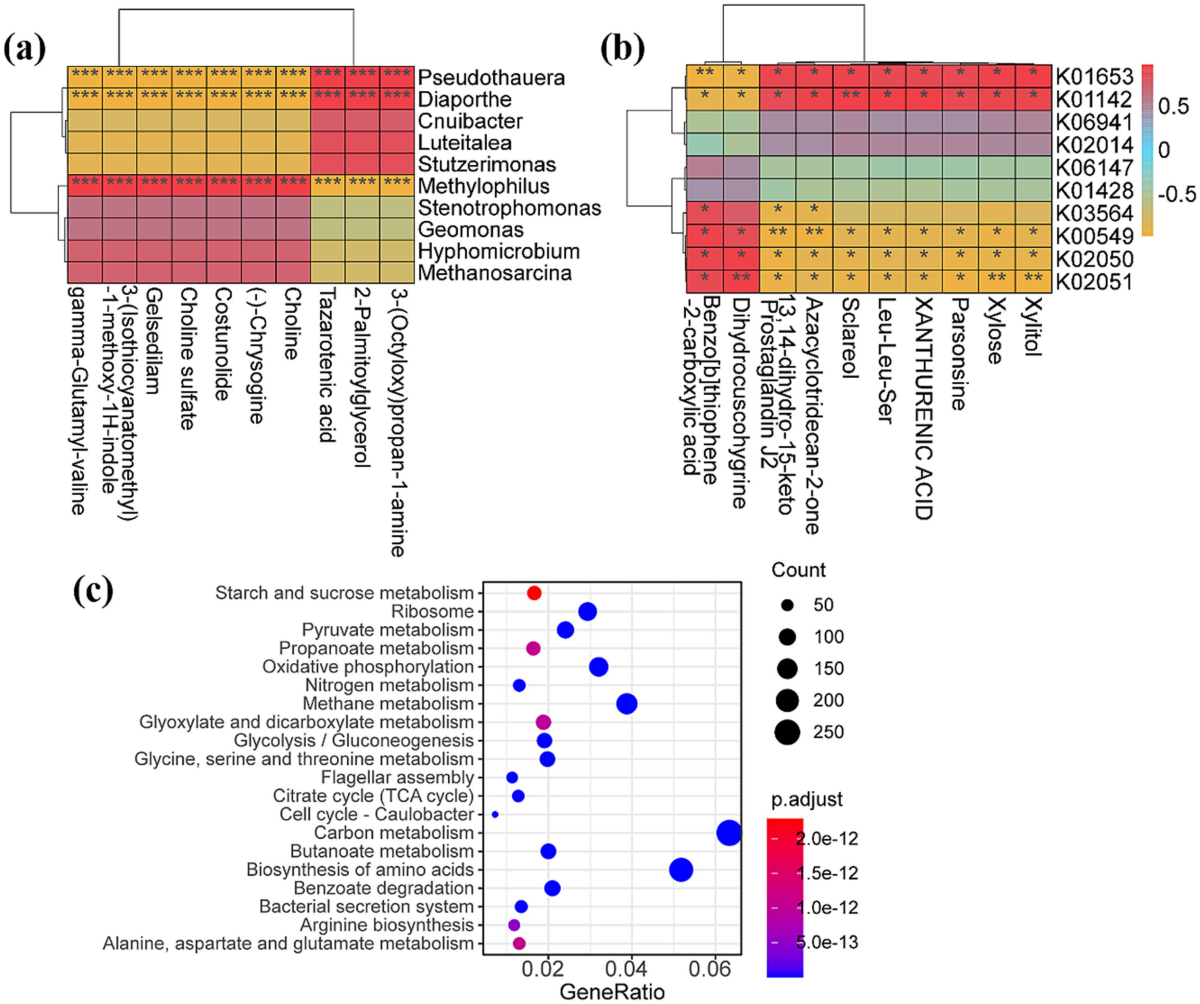
Integrated analysis of rhizosphere microbial and metabolic functions based on multi-omics data. **(a,b)** Correlation of the top 10 bacteria **(a)** and gene **(b)**, in terms of relative abundance, with the 10 differential metabolites. The vertical axis represents differential genera of bacteria or of enzymes (based on metagenomic sequences). The horizontal axis represents the differential metabolites. Red and green indicate positive and negative correlation, respectively. ***, **, and * are significant at the 0.001, 0.01 and 0.05 levels, respectively. **(c)** KEGG pathway enrichment analysis of functional genes in the rhizosphere soil under low temperature stress. The y-axis represents the significantly enriched metabolic pathways, and the x-axis represents the rich factor. The size of the dots indicates the number of differential genes, and the color depth represents the enrichment significance [−log₁₀ (*p* value)].

To extensively investigate the basic connections between microorganisms and metabolic functions in the rhizosphere micro-ecosystem of *P. clematidea* under LT stress, we conducted Spearman correlation analysis between significantly altered microbial genera (metagenomics) and differentially expressed metabolites (DEMs, untargeted metabolomics), resulting in an integrated heatmap (Figure 3a). The results revealed extensive and significant correlations between the rhizosphere soil microbiota and metabolites under LT versus HT treatments (|r| > 0.6, *p* < 0.001). Several core genera significantly enriched in the LT group, such as *Nocardiopsis*, *Sphingobium*, *Amycolatopsis*, and *Azoarcus*, showed strong positive correlations with a range of plant-microbe co-metabolites possessing potential stress-resistance functions. For instance, *Nocardiopsis* was significantly positively correlated with the terpenoid costunolide. Genera like *Sphingobium* and *Azoarcus* showed positive correlations with sulfur-containing compounds choline sulfate. Furthermore, several LT-enriched genera were positively correlated with the amino acid derivative gamma-glutamyl-valine.

Conversely, genera with higher relative abundance in the HT group, such as *Bradyrhizobium*, *Stenotrophomonas*, and *Methylophilus*, were mostly negatively correlated with the aforementioned metabolites. These genera showed stronger positive correlations with metabolites like gelsedilam and tazarotenic acid, whose contents decreased significantly in the LT environment. This suggests that the metabolic network co-occurring with the normal-temperature microbial community was substantially diminished under cold stress. The association analysis was conducted between key functional genes (KEGG Orthology, KO) annotated from the metagenomes and the DEMs (Figure 3b). The results indicated significant correlations between the abundance of microbial functional genes and the accumulation of various metabolites under LT stress, revealing the potential association between microbial metabolic genetic potential and the rhizosphere metabolome. These associations were primarily concentrated in key biological processes such as carbon source utilization, nitrogen metabolism, and stress response. The gene encoding transketolase (K01653) was positively correlated with intermediates of carbon source metabolism like xylose and xylitol. This enzyme is key to the pentose phosphate pathway (PPP) and its activity was positively linked to the accumulation of these compounds. Homologs or related regulatory units (e.g., K00549) of the *glnA* gene, encoding glutamine synthetase (K01915), showed close associations with the synthesis of the nitrogenous alkaloid dihydrocuscohygrine. The abundance of a gene encoding a sigma factor (K06941) was positively correlated with the accumulation of several plant/microbe-derived defensive compounds, including the terpenoid sclareol and the aromatic compound benzo[b]thiophene-2-carboxylic acid. The gene encoding beta-glucosidase (K01142) was closely associated with the degradation of oligosaccharides like cellobiose, and its activity is a rate-limiting step in cellulose degradation.

Further ORA pathway enrichment analysis was performed (Figure 3c). The results significantly revealed the profound impact of LT stress on rhizosphere metabolic activity, with DEMs highly concentrated in core microbial metabolic and biosynthetic pathways. The pathways related to amino acid metabolism (e.g., Biosynthesis of amino acids; alanine, aspartate and glutamate metabolism; arginine biosynthesis), carbon metabolism, and energy metabolism (e.g., carbon metabolism; glycolysis/gluconeogenesis; TCA cycle) formed the most significantly enriched core modules. This indicates that LT stress was coupled with a metabolic reprogramming signature in the rhizosphere microbial community, characterized by altered carbon and nitrogen resource allocation patterns and maintenance of energy homeostasis for environmental adaptation. Additionally, the enrichment of pathways such as biosynthesis of cofactors, methane metabolism, and benzoate degradation suggests that low temperatures may be associated with altered microbial utilization patterns for specific carbon sources (e.g., aromatic compounds).

## 4 Discussion

### 4.1 Low-temperature stress induces reorganization of carbon and nitrogen cycling in rhizosphere soil and enhances a cold-tolerant microbial community

The LT stress significantly altered the physicochemical properties and enzyme activities of the *P. clematidea* rhizosphere soil, indicating a profound impact on key processes of soil nutrient cycling. The soil enzymes serve as vital indicators of soil biological activity, and changes in their activities directly reflect the dynamics of soil nutrient cycling and the metabolic intensity of microorganisms (Luo et al., 2022; Wołejko et al., 2020). This study found that S-UE activity in the LT group was significantly higher than in the HT group (*p* < 0.05), suggesting that low temperature may have promoted urea hydrolysis and the nitrogen cycling process in the rhizosphere soil (Lai et al., 2025). Conversely, the activities of S-ALPT and S-CAT in the LT group were significantly lower than those in the HT group (*p* < 0.05).

The LT treatment noticeably changed the forms and distribution of soil nitrogen. The contents of NO₃^−^-N and NH₄^+^-N in the LT group rhizosphere soil were significantly higher than those in the HT group (*p* < 0.05), with a particularly sharp increase in ammonium-nitrogen. This phenomenon might be related to altered microbial activity under low temperatures; for instance, cold stress may have enhanced the activity of certain nitrifying and ammonifying bacteria or inhibited denitrification, leading to the accumulation of inorganic nitrogen (Zhu et al., 2020). However, opposing the trend of inorganic nitrogen, the TN content in the LT group was significantly lower than in the HT group (*p* < 0.05). Several non-mutually exclusive mechanisms may account for this apparent decline in TN despite inorganic N accumulation. While enhanced mineralization of soil organic nitrogen is consistent with the observed increase in S-UE activity and inorganic N pools, alternative or concurrent processes—such as gaseous nitrogen losses via denitrification or ammonia volatilization, increased leaching from the pot system under LT conditions, or shifts in microbial N immobilization—could also contribute to the observed pattern. Without direct measurement of nitrogen fluxes (e.g., denitrification rates or leachate N losses), the exact processes underlying the TN decline cannot be fully resolved.

Regarding the soil carbon pool and related indicators, the contents of TOC and SOM in the LT group were significantly lower than those in the HT group (*p* < 0.05). This may suggest that low temperatures accelerated the decomposition of soil organic matter and/or inhibited carbon input, leading to the depletion of the carbon pool (Su et al., 2022). Soil organic carbon is the primary energy source for microbial growth and activity, and its reduction could negatively affect the rhizosphere microbial community (Barré et al., 2018). Notably, MBC showed no significant difference between the two treatment groups (*p* > 0.05). This suggests that despite the decrease in TOC and organic matter, the microbial community maintained a relatively stable biomass, possibly by adjusting its composition or metabolic strategies (Tan and Luo, 2025).

As a strong environmental filter, low temperature has a significant impact on the organization of the microbial population. The NMDS analysis revealed a considerable separation between the LT and HT communities, but no significant difference in alpha diversity was noticed. These findings align with numerous studies indicating that environmental factors can significantly alter microbial community structure without necessarily changing diversity indices (He et al., 2022; Xiao et al., 2022). This suggests that low temperature did not reduce species richness but selectively filtered for cold-tolerant or psychrophilic taxa with specific functions. For instance, the relative abundances of *Nocardiopsis*, *Sphingobium*, *Azoarcus*, *Nocardioides*, and *Amycolatopsis* were significantly enriched in the LT group. Furthermore, these genera showed significant positive correlations with soil NH₄^+^-N and NO₃^−^-N contents. These genera are known for their diverse metabolic capabilities, including decomposing complex organic matter and participating in nitrogen cycling. Their enrichment likely represents a direct response to and involvement in the restructuring of carbon and nitrogen cycling described above (Parasar et al., 2024; Wang et al., 2023). Conversely, the relative abundances of several genera more abundant in the HT group, such as *Intrasporangium*, *Rhizophagus*, and *Bradyrhizobium*, were significantly reduced in the LT group. *Bradyrhizobium* is an important nitrogen-fixing bacterium, and its reduction under low temperature might impair the process of biological nitrogen fixation in the soil (Xiao et al., 2023). *Rhizophagus*, a genus of arbuscular mycorrhizal fungi (AMF), is crucial for plant nutrient acquisition and stress resistance; its decreased abundance could potentially exacerbate plant stress under low temperatures (Kandalgaonkar and Barvkar, 2025). These findings are consistent with our hypothesis that low-temperature stress selectively enriches cold-adapted microbial taxa with specific functional traits.

The correlation analysis between microbial taxa and soil physicochemical properties further confirmed the close links between community structure and soil environmental factors. Genera significantly enriched in the LT group, such as *Nocardiopsis*, *Azoarcus*, and *Amycolatopsis*, showed significant positive correlations with soil NH₄^+^-N and NO₃^−^-N contents, but negative correlations with TN content. This supports the hypothesis that these cold-tolerant genera are involved in nitrogen cycling processes under low temperatures, promoting the accumulation of inorganic nitrogen. Some members of the genus *Azoarcus* are known to perform nitrogen fixation or denitrification (Xing et al., 2021), and their enrichment at low temperatures could significantly influence soil nitrogen transformations. Additionally, the relative abundance of *Sphingobium* was negatively correlated with TOC and SOM content. This might imply that *Sphingobium* utilizes organic carbon more efficiently under cold conditions, or that its enrichment contributes to the depletion of organic carbon, thereby affecting the soil carbon pool (Wang L. et al., 2025; Xie et al., 2022). The genera more strongly associated with the HT group, such as *Bradyrhizobium*, were positively correlated with soil TN, which is consistent with its characteristic as a nitrogen-fixing bacterium likely maintaining higher nitrogen fixation activity at normal temperatures, contributing to TN accumulation (Zhong et al., 2024).

### 4.2 Alteration of the rhizosphere microbe–metabolite interaction network as a microecological strategy for cold adaptation

Under LT stress, the significant changes in the structure and functional metabolism of the microbial community, coupled with corresponding adjustments in soil metabolites within the *P. clematidea* rhizosphere, collectively establish a remodeled microbe-metabolite interaction network. This remodeling is identified as a key microecological strategy employed by *P. clematidea* to cope with LT stress. The metagenomic analysis revealed that low-temperature treatment significantly altered the metabolic potential of the rhizosphere microbial community. In nitrogen metabolism, the gene encoding glutamine synthetase (*glnA*) and the nitrate assimilation-related gene (*nasA*) both showed downregulation trends in the LT group. Glutamine synthetase is a key enzyme in the microbial nitrogen assimilation pathway (Torres-Beltrán et al., 2021); its downregulation may indicate that low temperature inhibited microbial assimilation and utilization of ammonium nitrogen, which aligns with the observed significant increase in NH₄^+^-N content in the LT group. Regarding carbon metabolism, the low-temperature treatment significantly affected several key pathways. Genes associated with the TCA cycle (*sdhA*, *sdhB*, *sucC*, *sucD*, *frdA*, *frdB*, and *mdh*), key glycolytic pathway genes (*GAPDH* and *gapA*), and acetate metabolism genes (*ACSS1_2*, *acs*) were generally upregulated in the LT group. The changes were also observed in the expression of genes related to isocitrate metabolism (*icd*, *IDH1*, and *IDH2*). This enrichment pattern in gene relative abundance suggests an elevated genetic potential for central carbon metabolism pathways, such as the TCA cycle and glycolysis, which could support increased energy generation for maintaining basic cellular functions and adapting to the low-temperature environment (Bartwal et al., 2013). However, as metagenomic gene abundance reflects metabolic potential rather than realized enzymatic activity, transcriptomic or proteomic validation would be necessary to confirm actual pathway upregulation. Such shifts in metabolic strategy represent a common adaptive mechanism for microorganisms facing environmental challenges (Liu et al., 2019).

The metabolomic results showed that low-temperature induction caused a global reprogramming of the rhizosphere soil metabolome. The increased levels of energy and cofactor-related metabolites, such as carbohydrates and vitamins, are consistent with the upregulation of carbon metabolic pathway genes observed in the metagenomic data, providing the material basis for sustaining energy-demanding basal metabolism under cold conditions. More importantly, a range of secondary metabolites with potential stress-resistance functions were significantly upregulated in the LT group. These included phenolic acids (e.g., Benzyl beta-primeveroside), flavonoids (e.g., Pectolinarigenin), and terpenoids (e.g., 13,21-Dihydroeurycomanone). These compounds are generally recognized to play important roles in stress responses in plants and microbes, such as scavenging reactive oxygen species, stabilizing cell membrane structures, and acting as signaling molecules (Ali et al., 2025; Sahu et al., 2022).

The correlation network analysis distinctly indicated intrinsic connections between particular microbial taxa and the co-occurrence of stress-resistant metabolites. The core genera enriched in the LT group, such as *Nocardiopsis*, *Sphingobium*, and *Amycolatopsis*, were significantly positively correlated with metabolites like costunolide and choline sulfate. This strongly suggests that under low-temperature stress, the cold-tolerant microorganisms enriched in the *P. clematidea* rhizosphere may not only synthesize these protective compounds themselves but could also potentially stimulate plant root exudation or co-participate in metabolic transformations. Consequently, a “protective metabolic microenvironment” conducive to resisting low-temperature-induced oxidative stress and membrane damage is established within the rhizosphere microzone (van Loo et al., 2018). However, as our analyses rely on correlation-based inference (Spearman‘s rank), the associations reported here do not establish causality. Metabolite accumulation could result from plant exudation, microbial biosynthesis, or combined processes. Targeted experimental validation (e.g., stable isotope probing or microbial isolation) is required to confirm specific functional relationships.

The association analysis between functional genes and metabolites provided potential molecular mechanisms for the aforementioned microbe-driven metabolic regulation. For instance, the positive correlation between a gene encoding a sigma factor and the accumulation of defensive compounds like sclareol indicates that low temperature might activate the expression of specific stress-response genes and the synthesis of secondary metabolites by regulating microbial transcription initiation (Cano-Ramirez et al., 2023). Meanwhile, pathway enrichment analysis showed that core metabolic pathways such as amino acid metabolism and the TCA cycle were the most concentrated areas for differential metabolites. This reflects a profound “metabolic reprogramming” signature within the rhizosphere microbial community under cold stress, likely aimed at reallocating resources to prioritize basic structural and energy metabolism essential for stress resistance and survival.

### 4.3 Holobiont perspective and the role of root exudates

Viewed through the holobiont framework, the coordinated shifts in rhizosphere microbial community structure, functional gene abundance, and metabolite profiles observed here suggest that *P. clematidea* may extend its cold-acclimation capacity by recruiting and maintaining a stress-mitigating microbiome. This microbial-mediated extension of plant phenotype likely provides a competitive advantage during range expansion into thermally challenging environments (Pérez-Llorca et al., 2023; Yang et al., 2022).

Root exudates play a pivotal role in regulating the rhizosphere microbial community and soil nutrient cycling (Parasar et al., 2024). Under LT stress, *P. clematidea* possibly alters the composition and quantity of its root exudates, thereby recruiting or selecting for specific microbial taxa to aid in its stress response. For instance, when plants are stressed, they release distinct blends of root exudates that play a key role in modulating the rhizosphere microbiome and soil nutrient cycling (Chai and Schachtman, 2022). The observed changes in microbial community structure, functional metabolism, and the availability of soil nitrogen, carbon, and phosphorus in this study collectively constitute a remodeling of the rhizosphere microbe-metabolite interaction network under low temperature. This remodeling likely facilitates *P. clematidea* adaptation to cold stress through the following pathways: (1) Cold-tolerant microorganisms maintain viability by utilizing limited resources more efficiently and adjusting their metabolic pathways; (2) The microbial community indirectly assists plants in acquiring essential nutrients by altering the forms and availability of soil nutrients; (3) Interactions between microorganisms and plants may induce cold tolerance or enhance the plant’s ability to withstand low-temperature stress. Future research could further incorporate transcriptomics and targeted metabolomics to thoroughly elucidate the specific interaction mechanisms between *P. clematidea* root exudates and the rhizosphere microbial community under low temperatures, as well as how these interactions precisely govern the cold tolerance or adaptation strategies of *P. clematidea*.

### 4.4 Study limitations

Several limitations of this study should be explicitly acknowledged. First, the experimental design did not include a bulk soil (plant-free) control. Without such a control, we cannot fully disentangle plant-mediated rhizosphere effects from direct temperature-driven shifts in soil microbial communities. Although the strong correlations between microbial genera and stress metabolites observed here suggest active plant–microbe interactions, future experiments incorporating plant-free controls are needed to substantiate these interpretations. Second, all microbial and metabolic interpretations are based on correlation analyses (Spearman’s rank). Therefore, causal relationships between specific microbial taxa and metabolite accumulation cannot be definitively established without targeted experimental validation (e.g., stable isotope probing or microbial isolation). The associations reported here should be viewed as hypothesis-generating and not as evidence of direct functional regulation. Third, metagenomic gene abundance reflects genetic potential rather than realized metabolic activity. Although we interpret gene abundance patterns as indicative of functional potential, verification through metatranscriptomic or metaproteomic approaches would be necessary to confirm actual changes in pathway activity. Fifth, direct nitrogen flux measurements were not performed in this study. Consequently, the exact processes underlying the observed TN decline—whether enhanced mineralization, gaseous N losses, or leaching—cannot be fully resolved. Despite these constraints, our integrated multi-omics approach provides a comprehensive and hypothesis-generating framework that can guide future targeted investigations into the rhizosphere mechanisms underlying plant cold adaptation.

## 5 Conclusion

This study employs an integrated multi-omics analysis to elucidate the adaptive response mechanisms of the rhizosphere soil micro-ecosystem of the invasive plant *P. clematidea* under low-temperature induction. The results demonstrated that 5 °C low-temperature stress did not reduce microbial diversity but, acting as a powerful environmental filter, significantly drove a directional succession of the microbial community structure towards cold-tolerant functional taxa such as *Nocardiopsis* and *Sphingobium*. This shift was associated with profoundly restructured rhizosphere nutrient cycling processes: enhanced mineralization of soil organic nitrogen coupled with weakened nitrogen assimilation genetic potential was linked to the accumulation of inorganic nitrogen, while enriched carbon metabolic pathway genes were associated with indicators of accelerated energy production. Additionally, LT stress was associated with altered rhizosphere metabolome composition, including phenolic acids and flavonoids, which exhibited notable positive associations with the enriched cold-tolerant bacteria. Thus, the current study elucidates a micro-ecological mechanism based on the synergistic interaction among “microbial community succession—functional gene expression—rhizosphere metabolic network remodeling.” This coordinated mechanism likely enhances the adaptability of *P. clematidea* to low-temperature stress. However, these findings were obtained under controlled pot-based conditions, and direct extrapolation to field-scale invasion success requires caution. Future field-based studies are essential to validate the ecological significance of these mechanisms in natural settings.

## Data Availability

The datasets presented in this study can be found in online repositories. The names of the repository/repositories and accession number(s) can be found in the article/Supplementary material.

## Footnotes

1
https://www.bioincloud.tech/
