# Supplementary material for: Integrated metagenomic and metabolomic insights into microbial metabolic reprogramming in the rhizosphere of the invasive plant Praxelis clematidea under low-temperature stress
Source: Front Microbiol. 2026 Jul 15;17:1852122. doi: 10.3389/fmicb.2026.1852122 (PMC13416216; doi:10.3389/fmicb.2026.1852122)
Supplement: Supplementary file 1 [file Supplementary_file_1.pdf]

Appendix A: PCA diagram of quality control sample

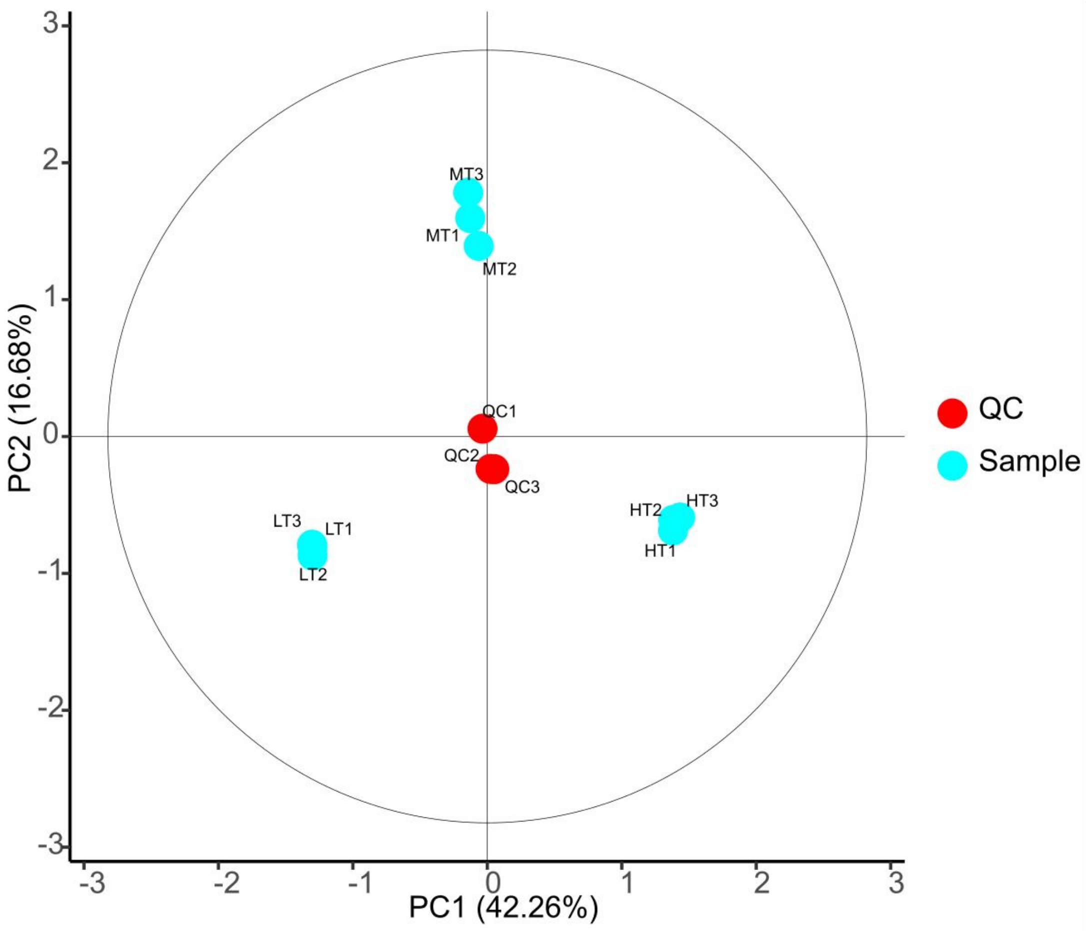

Figures S1 PCA diagram of quality control sample

Note: The red dot is the quality control sample point (QC sample) after correction, and the blue dot is the test sample.

Appendix B: Plot of the OPLS-DA permutation test

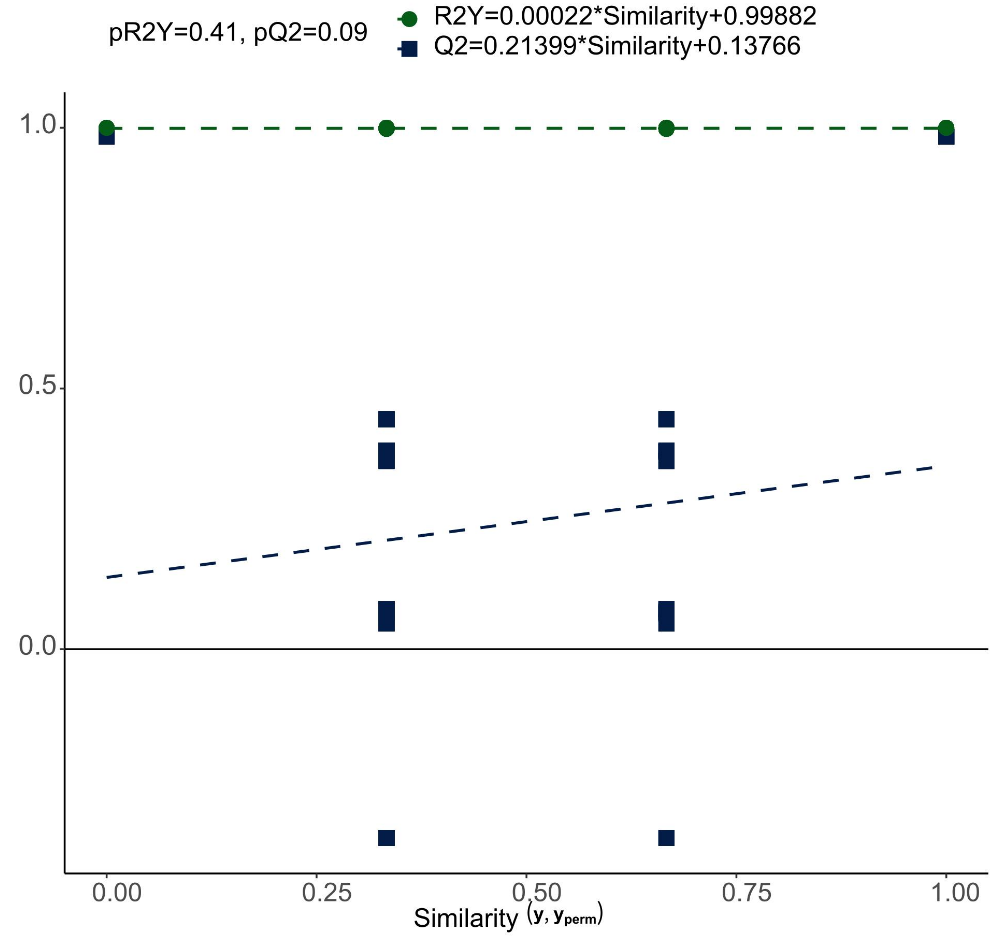

Figures S2 OPLS-DA permutation test plot

Note: The x-axis represents the similarity between the permuted group labels and the true group labels, usually expressed as the proportion of identical labels (range 0–1). When OPLS-DA is applied to more than three groups, the groups need to be converted into ordinal values (so the model becomes an OPLS regression, OPLS-R); in that case, the similarity is given by the correlation coefficient and can be negative (range -1 to 1). The y-axis shows the R^2Y and Q^2 values for each permutation. The values at x = 1 correspond to the Q^2 and R^2Y of the final model. The two straight lines in the plot are the regression lines for R^2Y and Q^2, respectively, and the regression equations are provided in the legend.
